# Treatment of sarcopenia: the road to the future

**DOI:** 10.1002/jcsm.12386

**Published:** 2019-01-29

**Authors:** John E. Morley

**Affiliations:** ^1^ Division of Geriatric Medicine Saint Louis University School of Medicine St. Louis USA

**Keywords:** Sarcopenia, muscle, treatment

This year, two new consensus conferences on the diagnosis and management of sarcopenia have been published.^1^, ^2^ Both confirm the need to screen for sarcopenia in older persons. Suggested screening approaches are the SARC‐F,^3^, ^4^ the Ishii screening test,^5^, ^6^ or grip strength. It should be recognized that grip strength was suggested for screening by one consensus group^1^ and as part of the diagnosis by the other.^2^ Measuring mid‐calf muscle circumference improves the sensitivity and specificity of the SARC‐F when it is compared with the consensus definitions.^7^, ^8^, ^9^

A number of consensus definitions for sarcopenia have been developed.^2^, ^11^, ^12^, ^13^ All require either functional impairment (slow walking speed) or grip strength together with a low muscle mass. While the persons diagnosed by any of these definitions overlap, they all have different sensitivity and specificity when compared with one another or functional outcomes due to the different cut‐off points.^14^, ^15^ The Asian Group made it clear that cut‐offs are very different for persons with Asian ethnicity compared with Europeans.^13^ [These definitions have led to the International Classification of Disease (10th edition) to recognize sarcopenia as an independent condition (M62.84)].^16^, ^17^

There are a number of different methods available to measure lean body mass including air displacement plethysmography, bioelectrical impedance analyses, dual‐energy X‐ray absorptiometry, and ultrasound.^18^, ^19^, ^20^, ^21^ Each of these methods has been demonstrated to have problems in accurately determining muscle mass.^22^ Recently, D_3_‐creatine dilution has been demonstrated to be more accurate in measuring muscle mass^23^ and more strongly related to physical performance.^22^

While age‐related sarcopenia is considered to be primary sarcopenia, a number of disease states, for example, diabetes mellitus,^24^, ^25^ male hypogonadism,^26^, ^27^ and chronic obstructive pulmonary disease^28^ can produce secondary sarcopenia. Cachexia is a complex metabolism disorder leading to anorexia, muscle wasting, and loss of fat.^29^ The Glasgow Prognostic Score (low serum albumin and elevated C‐reactive protein) can be used to distinguish secondary sarcopenia from cachexia.^30^

The advent of patient‐centred (P4) care has increased attention to the fact that different molecular changes can result in the need to have different therapeutic approaches to similar conditions such as sarcopenia^31^, ^32^ (Table 1). In this issue of the journal, Riuzzi *et al*.^33^ highlight that sarcopenia can result from a variety of molecular changes resulting in changes in myofibre metabolism and alterations in satellite cell properties. Abnormalities in these pathways can be due to insulin growth factor‐1/insulin receptors, activin (myostatin) receptors, tropomysin receptor, kinase C receptors (neurotrophin and G‐protein receptors), a variety of cytokines, and testosterone through activation of β‐catenin.^34^, ^35^, ^36^, ^37^, ^38^ Thus, in the long run, the ideal treatment of sarcopenia will involve identification of the aberrant molecular pathway and the possible hormone causing this imbalance.

**Table 1:** Patient‐centred approach to management of sarcopenia

Early identification	Primary prevention	Secondary prevention	Tertiary prevention
SARC‐F or ISHII screening test	Exercise	Resistance exercise	Physical therapy
	Adequate protein diet	Low‐protein diet: leucine‐enriched essential amino acids or methyl hydroxy butyrate supplementation	Occupational therapy
	In ALL hospitalized: aggressive resistance exercise (include intensive care unit)	Male hypogonadism: testosterone	If dysphagia: speech therapy
		If falling: use CDC STEADI or F3ALLS approach	Provide adequate protein intake
		If low 25(OH) vitamin D—1000 IU vitamin D	Optimal treatment of COPD; CHF and diabetes mellitus
			Exclude cachexia: elevated CRP + low protein
			Exclude protein energy malnutrition (anorexia or malabsorption)
			‐Look for treatable causes
			‐Caloric supplement
			‐Future: anamorelin
			Future: antibodies to myostatin

At present, the treatment of sarcopenia is focused on resistance exercise.^1^ The use of leucine essential amino acids and/or β‐hydroxybutyrate has not been clearly established but would seem a reasonable adjunct in persons with low protein intake.^39^, ^40^, ^41^, ^42^, ^43^, ^44^, ^45^ Drugs that have potential to treat sarcopenia include testosterone and anabolic steroids,^46^, ^47^, ^48^ myostatin antibodies,^49^, ^50^ activin receptor antibodies,^51^ and the ghrelin agonist, anamorelin.^52^ There is also interest in the role of beta‐blockade,^53^ some angiotensin‐converting enzyme inhibitors,^54^ and sarconeos, which activates the MAS (angiotensin‐1) receptor.^55^ A recent study suggested that metformin may improve mobility in persons with diabetes mellitus.^56^ Still highly experimental but likely to play a role in the future management of sarcopenia are CRISPR techniques^57^ and possibly stem cell therapy.^58^

Sarcopenia is a major cause of physical frailty^59^, ^60^, ^61^ and falls^62^, ^63^ in older persons. As at present, there is a simple therapy—aggressive resistance exercise—when sarcopenia is detected early, it seems reasonable to screen older persons and those with diabetes for sarcopenia and frailty using the Rapid Geriatric Assessment tool^64^, ^65^, ^66^, ^67^ and begin secondary prevention as early as possible. The SarQOL can be utilized to measure an improvement of health‐related quality of life in these persons.^68^ A recent study demonstrated that an intense level of physical exercise in hospital patients can prevent the muscle and functional loss that occurs in hospitalized patients.^69^

## Conflict of interest

None declared.
